# Epidemiological characteristics of *Vibrio parahaemolyticus* outbreaks, Zhejiang, China, 2010–2022

**DOI:** 10.3389/fmicb.2023.1171350

**Published:** 2023-06-28

**Authors:** Lili Chen, Jikai Wang, Jiang Chen, Ronghua Zhang, Hexiang Zhang, Xiaojuan Qi, Yue He

**Affiliations:** Department of Nutrition and Food Safety, Zhejiang Provincial Center for Disease Control and Prevention, Hangzhou, China

**Keywords:** foodborne disease outbreak, *Vibrio parahaemolyticus*, ready-to-eat food, serotype, seafood

## Abstract

**Background:**

*Vibrio parahaemolyticus* is one of the most common foodborne pathogens and poses a significant disease burden. The purpose of the study was to elucidate the epidemiological characteristics of *Vibrio parahaemolyticus* outbreaks in Zhejiang Province, and provide insights for the targeted prevention and control of foodborne diseases.

**Methods:**

Descriptive statistical methods were utilized to analyze the data on *Vibrio parahaemolyticus* outbreaks reported by all Centers for Disease Control and Prevention (CDCs) through Foodborne Disease Outbreaks Surveillance System (FDOSS) in Zhejiang Province from 2010 to 2022.

**Results:**

From 2010 to 2022, a total of 383 outbreaks caused by *Vibrio parahaemolyticus* were reported by 90 CDCs in 11 prefectures of Zhejiang Province, resulting in 4,382 illnesses, 326 hospitalizations and 1 death. The main symptoms of the outbreak-related cases were diarrhea (95.18%), abdominal pain (89.23%), nausea (55.64%), vomiting (50.57%), fever (24.21%), etc. The outbreaks occurring between July and September accounted for 77.54% of all outbreaks (297 out of 383). Outbreaks associated with restaurants accounted for the majority (57.96%, 222/383) of all outbreaks, followed by those linked to staff canteens (15.40%, 59/383) and rural banquets (11.23%, 43/383). 31.85% of all outbreaks were associated with the consumption of aquatic products, while ready-to-eat foods such as Chinese cold dishes and cooked meat products accounted for 12.53% of all outbreaks. Serotype O3:K6 (81.94%, 118/144) was the predominant serotype responsible for outbreaks from 2010 to 2020, while serotype O10:K4 (57.89%, 33/57) was the predominant serotype from 2021 to 2022.

**Conclusion:**

In-depth and comprehensive analysis of long-term surveillance data on *Vibrio parahaemolyticus* outbreaks is essential to gain insight into the epidemiological characteristics, identify long-term patterns and recent trends, and enable governments to prioritize interventions and develop targeted policies to mitigate such outbreaks.

## 1. Introduction

*Vibrio parahaemolyticus* is a gram-negative autochthonous bacterium that can be found in temperate and tropical marine and coastal waters worldwide, and it is one of the most prevalent foodborne pathogens (Martinez-Urtaza and Baker-Austin, 2020). It typically results in self-limiting gastroenteritis, but can be fatal in immunocompromised patients or those with pre-existing conditions such as liver disease or diabetes (Yeung and Boor, 2004). In 1950, *Vibrio parahaemolyticus* was first isolated in Japan. Since the worldwide prevalence of O3:K6 strain in 1996, *Vibrio parahaemolyticus* has gradually emerged as a major global foodborne pathogen (Hara-Kudo et al., 2012). *Vibrio parahaemolyticus* is the leading cause of bacterial gastroenteritis associated with seafood consumption in numerous countries, including the United States (Scallan et al., 2011; Newton et al., 2012) and various Asian countries (Sen et al., 2007; Park et al., 2018). *Vibrio parahaemolyticus* infections can lead to significant medical expenses. Seafood contaminated with *Vibrio parahaemolyticus* causes an estimated of $21 million in annual health-related costs in the United States (Ralston et al., 2011). In China, *Vibrio parahaemolyticus* is a major pathogen responsible for bacterial foodborne outbreaks (Li et al., 2020), particularly in coastal regions such as Zhejiang Province and Shanghai (Qi et al., 2019; Chen et al., 2022). According to a study conducted in Shanghai (Luo et al., 2019), the annual incidence rate of *Vibrio parahaemolyticus* gastroenteritis was estimated at 183 cases per 100,000 individuals, which is significantly higher than that reported by studies from both the United States (12 per 100,000) (Scallan et al., 2011) and Japan (65 per 100,000) (Kubota et al., 2011).

*Vibrio parahaemolyticus* grows preferentially in warm (>15°C), low-salinity marine water (<25 ppt NaCl) (Martinez-Urtaza and Baker-Austin, 2020). Studies have shown that the pathogen dies or at least becomes inactive at temperatures below 10°C (Parveen et al., 2008; Shen et al., 2009; Fernandez-Piquer et al., 2011). Cruz et al. (2015) found that the presence and numbers of *Vibrio parahaemolyticus* observed in summer months were significantly higher than in winter months (*p* < 0.001). Xu et al. (2016) also observed a significant seasonal variation in the density of *Vibrio parahaemolyticus*, with mean levels of 16.5 MPN/g and 5.0 MPN/g during summer and winter, respectively. Therefore, the concentration of *Vibrio parahaemolyticus* in freshly caught seafood can be reduced by controlling the temperature. Furthermore, according to a study, the populations of *Vibrio parahaemolyticus* in oysters demonstrated rapid growth from non-detectable (<3 MPN/g) to 4.72, 5.04, 5.72, and 6.66 logMPN/g at temperatures of 16°C, 20°C, 26°C and 32°C, respectively, after being exposed to contaminated artificial seawater for a period of 32 h. Thus, when seafood is not properly preserved after harvest, *Vibrio parahaemolyticus* may increase from initially low concentrations to dangerously high concentrations. To prevent the growth of this pathogen, it is recommended by the Japanese Ministry of Health, Labour, and Welfare (MHLW) that seafood handlers maintain oysters at temperatures below 10°C during distribution and storage; similar policies are also implemented in the United States, Australia, and New Zealand (Ndraha et al., 2020).

Consumption of raw or improperly cooked seafood is a common cause of *Vibrio parahaemolyticus* outbreaks. Taylor et al. (2018) reported the largest outbreak of *Vibrio parahaemolyticus* associated with consumption of raw oysters experienced in Canada during the summer of 2015. McLaughlin et al. (2005) reported an outbreak that occurred on a cruise ship in Alaska and found that consumption of raw oysters was the only significant predictor of disease. There was also an outbreak in Mexico, where all cases were attributed to the consumption of raw or undercooked shrimp (Héctor et al., 2006). Additionally, cross-contamination serves as a common contributing factor to *Vibrio parahaemolyticus* outbreaks. Martinez-Urtaza et al. (2016) reported a *Vibrio parahaemolyticus* outbreak caused by the cross-contamination of cooked shrimp with ice used for cooling purposes. Jung (2018) reported an outbreak in Korea caused by cross-contamination between kimbap and squid due to the sharing of cutting board and knife. These findings highlight the critical significance of preventing cross-contamination, as even cooked food can be contaminated by other foods carrying *Vibrio parahaemolyticus* through cross-contamination. As ready-to-eat foods are not subjected to heat treatment prior to consumption, contamination with *Vibrio parahaemolyticus* may lead to the incidence of foodborne diseases. Several studies have revealed the presence of *Vibrio parahaemolyticus* in commonly consumed ready-to-eat foods (such as Chinese cold dishes and cooked meat products) in China (Xie et al., 2016; Pang et al., 2019; Li et al., 2020; Xie et al., 2020). Outbreaks resulting from the consumption of previously mentioned ready-to-eat food contaminated with *Vibrio parahaemolyticus* have also been reported (Li et al., 2021; Wu L. Z. et al., 2022; Wu G. J. et al., 2022).

Since 2010, we have accumulated significant amounts of data on both foodborne disease outbreaks and sporadic cases through the utilization of two surveillance systems: the Foodborne Disease Outbreaks Surveillance System (FDOSS) and the Foodborne Disease Surveillance and Reporting System (FDSRS). By analyzing these surveillance data, we can obtain the epidemiological characteristics of foodborne diseases caused by specific pathogens, such as *Vibrio parahaemolyticus*, *Salmonella*, etc. A study (Qi et al., 2022) of 3,022 sporadic cases of *Vibrio parahaemolyticus* in Zhejiang Province from 2016 to 2020 revealed distinct seasonal distribution patterns, with the highest incidence occurring during summer and peaking in August. Furthermore, aquatic products were identified as the most frequently reported suspicious food, followed by cooked meat products. However, these suspected foods have not been confirmed through epidemiological investigations and further details are currently unavailable.

The aim of this study was to analyze the *Vibrio parahaemolyticus* outbreak data in Zhejiang province from 2010 to 2022, which were obtained from FDOSS. A foodborne disease outbreak is defined as the occurrence of two or more cases of a similar illness resulting from the ingestion of a common food, or if the food vehicle was undecided, sharing a common meal or food facility (Wu et al., 2014). These outbreak data were confirmed by CDCs through epidemiological investigation and included details such as the time and location of occurrence, etiology, food, setting, contributing factors, serotypes and symptoms of cases. Therefore, analysis of these data can be more accurately and comprehensively to obtain epidemiological characteristics, and provide important information for future intervention and prevention of foodborne diseases caused by *Vibrio parahaemolyticus*.

## 2. Materials and methods

### 2.1. Diagnostic criteria for *Vibrio parahaemolyticus* outbreaks

The diagnostic criteria for *Vibrio parahaemolyticus* outbreaks were based on epidemiological characteristics, clinical manifestations, and laboratory diagnosis (Ministry of Health of the People’s Republic of China, 1996). Outbreaks that did not meet the criteria were not reported as *Vibrio parahaemolyticus* outbreaks to the Foodborne Disease Outbreaks Surveillance System (FDOSS). The etiology of the outbreak is considered confirmed if *Vibrio parahaemolyticus* was isolated from the food involved or equipment or utensils used or a simultaneous serotype of *Vibrio parahaemolyticus* was detected from stool samples or vomit of multiple cases. If *Vibrio parahaemolyticus* was the only reported etiology, outbreaks were included in the analysis, while outbreaks caused by multiple pathogens were excluded from our study.

### 2.2. Standard laboratory protocol for *Vibrio parahaemolyticus* outbreak investigation

Isolation and identification of *Vibrio parahaemolyticus.* The isolation and identification of *Vibrio parahaemolyticus* in food in each outbreak were conducted in accordance with the recommendations of *Vibrio parahaemolyticus* Test (GB 4789.7-2013), the national standard for food safety. The experimental procedure is outlined as follows. A sample of 25 g (mL) was taken in aseptic procedure and homogenized with 225 mL of 3% sodium chloride alkaline peptone water (1:10). The sample was incubated in a 36°C ± 1°C incubator for 8–18 h, after which the TCBS plate or Vibrio chromogenic medium plate was streaked and cultured at 36°C ± 1°C for 18–24 h. The detection of *Vibrio parahaemolyticus* in fecal samples was conducted according to the inspection procedures in the Foodborne Disease Surveillance Workbook. Specimens include fresh fecal or anal swabs, or those transferred to Cary-Blair transport medium. A small amount of feces were collected from sterile swabs and the swabs were put into 3% of sodium chloride alkaline peptone water, or the feces swabs transferred to Cary-Blair transport medium were put into alkaline peptone water and cultured at 36°C ± 1°C for 12–16 h. Then, the TCBS plate or Vibrio chromogenic medium plate was streaked and cultured at 36°C ± 1°C for 18–24 h. Colonies should be picked or marked as soon as possible (no more than 1 h) after the TCBS plate is removed from the incubator. The characteristics of typical *Vibrio parahaemolyticus* on vibrio chromogenic medium were determined according to the product instructions. Three or more suspicious colonies were selected and streaked with 3% of sodium chloride tryptone soybean AGAR plate, and cultured at 36°C ± 1°C for 18–24 h. Subsequently, *Vibrio parahaemolyticus* was preliminarily identified based on the results of oxidase and halophilic tests as well as other experimental procedures. ONPG test was performed on 3% of sodium chloride ferric triose AGAR overnight culture. A complete loop of fresh culture was carefully selected and inoculated into 0.25 mL of 3% of sodium chloride solution. One drop of toluene was added in a ventilated environment, followed by shaking before placing the culture in a water bath at 37°C for 5 min. Afterward, add 0.25 mL ONPG solution and incubate the culture at 36°C ± 1°C for a duration of 24 h. Positive results will manifest as yellow coloration while negative results will remain unchanged after being observed for up to 24 h. Finally, *Vibrio parahaemolyticus* was identified using a biochemical identification kit (Bio Mérieux, Lyon, France) or an automatic microbial biochemical identification system (Bio Mérieux, Lyon, France).

Serotyping of *Vibrio parahaemolyticus.* Confirmed *Vibrio parahaemolyticus* isolates were serotyped by slide agglutination using commercial antisera according to the manufacture’s instruction (Denka Seiken, Tokyo, Japan).

### 2.3. Data source

FDOSS passively collects foodborne outbreak data from all CDCs in 11 prefectures of Zhejiang Province. From 2010 and 2022, outbreak reports were available from 11 surveillance prefectures including Hangzhou, Ningbo, Wenzhou, Jiaxing, Huzhou, Shaoxing, Jinhua, Quzhou, Zhoushan, Taizhou and Lishui. CDCs are responsible for investigating outbreaks within their jurisdiction and reporting epidemiological reports to the FDOSS according to uniform standards. Information collected for each outbreak included the time and location of occurrence, etiology, food, setting, number of cases, hospitalizations and deaths, contributing factors, serotypes, symptoms of cases and other details. The contributing factors mainly included improper food processing, cross contamination between raw and cooked food, improper food storage, contamination or deterioration of raw materials, contamination of equipment, contamination of personnel.

### 2.4. Statistical analysis

The data utilized in this study were sourced from FDOSS spanning the years 2010 to 2022 and subjected to analysis using Excel 2013. All variable values were reported as counts or proportions (%). The number and proportion of outbreaks, illnesses, hospitalizations, and deaths by setting and food were calculated. In addition, we conducted analyses on regional and temporal distribution as well as contributing factors, serotypes, and symptoms. The *per capita* incidence rate of outbreaks in Zhejiang Province from 2010 to 2022 was calculated based on population data sourced from the Statistical Yearbook of Zhejiang Province (Zhejiang Provincial Bureau of Statistics, 2010–2022). The population in 2016 was selected as the denominator due to its position at the midpoint of the years analyzed.

## 3. Results

### 3.1. General characteristics

**Figure 1:**
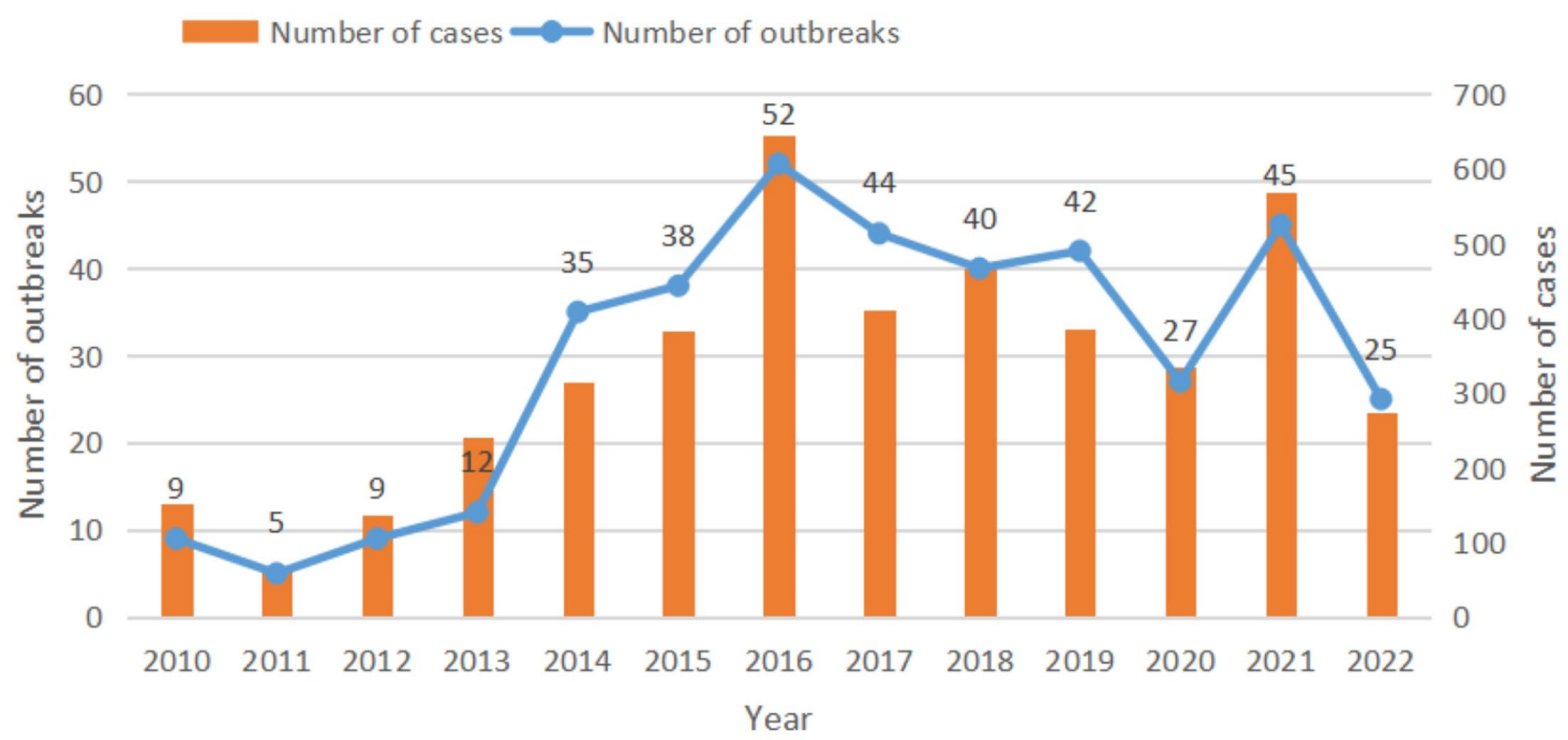
The number of outbreaks and cases of *Vibrio parahaemolyticus* outbreaks, 2010–2022, Zhejiang Province.

From 2010 to 2022, a total of 383 outbreaks caused by *Vibrio parahaemolyticus* were reported by 90 CDCs in 11 prefectures of Zhejiang Province, resulting in 4,382 illnesses, 326 hospitalizations and 1 death. The median number of cases in all outbreaks was 8 (range: 2–90). The number of outbreaks increased from 2010 to 2016, peaked in 2016, and began to decrease in 2017 (Figure 1). There was little difference in the number of outbreaks from 2017 to 2019, but the number of outbreaks decreased significantly in 2020 and 2022. During the period of 2010–2013, an average of 8.8 outbreaks and 147.8 cases occurred annually, while during the period of 2014–2022, an average of 38.7 outbreaks and 421.2 cases occurred annually. The incidence rate of outbreak-related cases varied from 1.1 to 11.6 cases per million population during the 13-year period. The mortality rate associated with outbreaks was 0.018 per 1 million population. The main symptoms of the outbreak-related cases were diarrhea (95.18%, 4171/4382), abdominal pain (89.23%, 3910/4382), nausea (55.64%, 2438/4382), vomiting (50.57%, 2216/4382), fever (24.21%, 1061/4382), etc. In all reported outbreaks, the maximum incubation period was 37 h and the minimum was only 1 h.

### 3.2. Regional distribution

Outbreaks of *Vibrio parahaemolyticus* were reported in 11 prefectures (Table 1), with Hangzhou recording the highest number of outbreaks (63 outbreaks), while Quzhou had the lowest number of outbreaks (13 outbreaks). The regions of Zhoushan, Lishui, and Huzhou had the highest incidence rates of outbreak-associated cases per 1 million population at 23.37, 12.95, and 7.85, respectively.

**Table 1:** Regional distribution of *Vibrio parahaemolyticus* outbreaks, 2010–2022, Zhejiang Province.

Region	Outbreaks		Cases		Incidence rates of cases
	*n*	%	*n*	%	1,000,000
Hangzhou	63	16.45	652	14.88	5.56
Wenzhou	46	12.01	672	15.34	5.67
Ningbo	40	10.44	633	14.45	6.22
Zhoushan	39	10.18	350	7.99	23.37
Jiaxing	37	9.66	430	9.81	7.21
Taizhou	35	9.14	256	5.84	3.26
Shaoxing	34	8.88	282	6.44	4.37
Lishui	33	8.62	360	8.22	12.95
Jinhua	27	7.05	279	6.37	3.94
Huzhou	16	4.18	301	6.87	7.85
Quzhou	13	3.39	167	3.81	6.02
Total	383	100.00	4382	100.00	6.09

### 3.3. Temporal distribution

**Figure 2:**
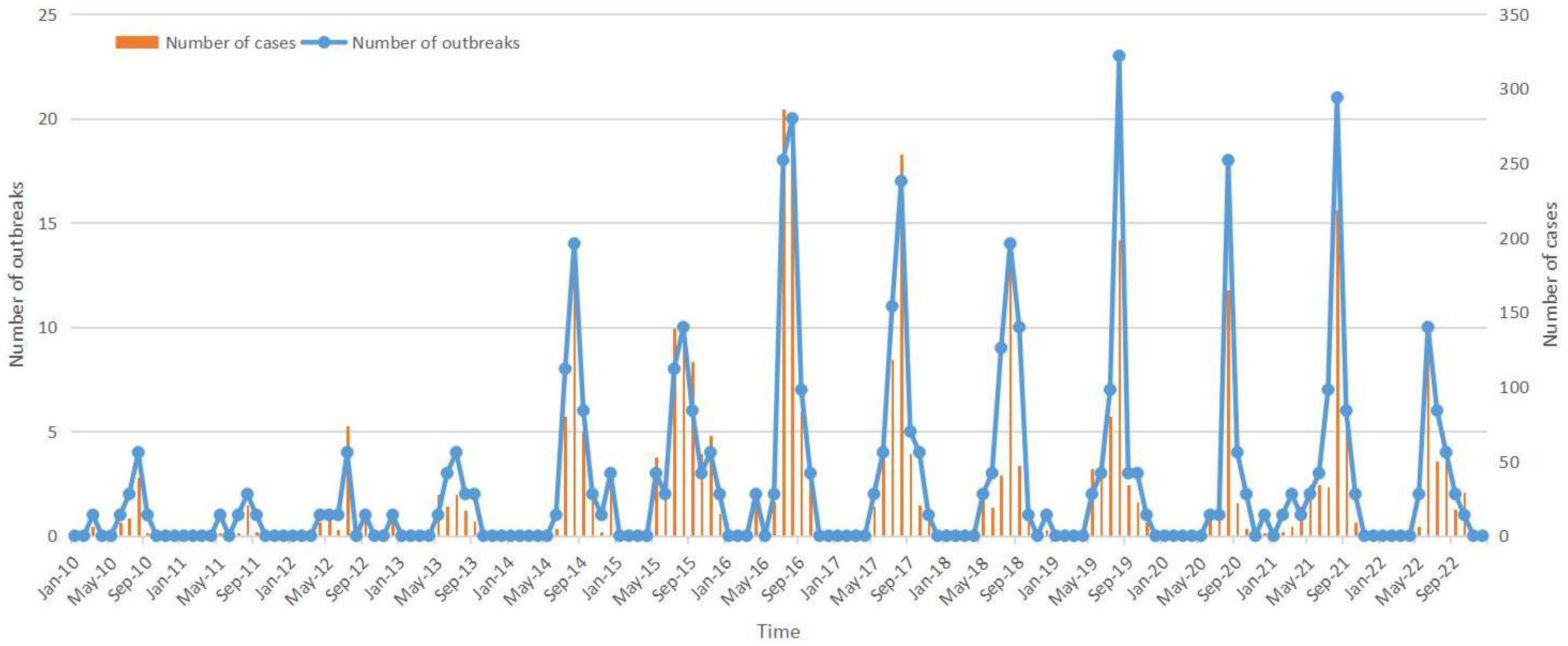
Temporal distribution of *Vibrio parahaemolyticus* outbreaks, 2010–2022, Zhejiang Province.

The incidence of *Vibrio parahaemolyticus* outbreaks exhibits a distinct seasonality, with the peak period occurring between July and September (Figure 2). The number of *Vibrio parahaemolyticus* outbreaks began to increase from May (14 outbreaks), and decreased rapidly after reaching a peak in August (151 outbreaks), with obvious seasonal characteristics. The number of outbreaks from July to September accounted for 77.54% of all outbreaks (297 outbreaks, 297/383), and the number of cases accounted for 78.5% (3440 cases, 3440/4382) of the total.

### 3.4. Setting

*Vibrio parahaemolyticus* outbreaks were most commonly reported in restaurants, accounting for 57.96% of all outbreaks, followed by staff canteens (15.40%) and rural banquets (11.23%) (Table 2). The school canteens had the highest number of cases per outbreak (27 cases, 163/6), followed by staff canteens (15 cases, 889/59), rural banquets (13 cases, 532/43) and restaurants (11cases, 2435/222). The largest recorded *Vibrio parahaemolyticus* outbreak occurred in a restaurant, resulting in 90 cases. Additionally, two other large outbreaks of 69 cases each took place at a restaurant and a high school canteen.

**Table 2:** Setting distribution of *Vibrio parahaemolyticus* outbreaks, 2010–2022, Zhejiang Province.

Settings	Outbreaks		Cases		Hospitalizations		Deaths	
	*n*	%	*n*	%	n	%	*n*	%
Restaurant	222	57.96	2435	55.57	138	42.33	1	100
Staff canteen	59	15.40	889	20.29	85	26.07	0	0
Rural banquet	43	11.23	532	12.14	53	16.26	0	0
Household	34	8.88	190	4.34	20	6.13	0	0
Street stall	9	2.35	96	2.19	4	1.23	0	0
School canteen	6	1.57	163	3.72	17	5.21	0	0
Fast food restaurant	4	1.04	37	0.84	5	1.53	0	0
Deliver meals	4	1.04	31	0.71	0	0.00	0	0
Other	2	0.52	9	0.21	4	1.23	0	0
Total	383	100.00	4382	100.00	326	100.00	1	100

### 3.5. Food

The food vehicle was confirmed in 53.26% (204/383) of all outbreaks, and 46.47% (178/383) of the food vehicles were single foods (Table 3). The food that caused the most *Vibrio parahaemolyticus* outbreaks were aquatic products (31.85%), including cooked aquatic products (28.72%) and raw aquatic products (3.13%). Cooked aquatic products mainly included shrimp (31 outbreaks), shellfish (30 outbreaks), fish (23 outbreaks), and crab (8 outbreaks). Raw aquatic products were mainly crabs marinated in salt or wine (8 outbreaks). The second highest number of outbreaks was attributed to ready-to-eat foods (12.53%), which included Chinese cold dishes (7.05%) and cooked meat products (5.48%).

**Table 3:** Food distribution of *Vibrio parahaemolyticus* outbreaks, 2010–2022, Zhejiang Province.

Food	Outbreaks		Cases		Hospitalizations		Deaths	
	Number	%	Number	%	Number	%	Number	%
**Aquatic products**	122	31.85	1,283	29.28	75	23.01	0	0.00
Cooked aquatic products	110^a^	28.72	1217	27.77	69	21.17	0	0.00
Raw aquatic products	12	3.13	66	1.51	6	1.84	0	0.00
**Ready-to-eat foods**	48	12.53	641	14.63	92	28.22	0	0.00
Chinese cold dish	27	7.05	393	8.97	24	7.36	0	0.00
Meat and meat products	21	5.48	248	5.66	68	20.86	0	0.00
Mixed dishes	26	6.79	273	6.23	16	4.91	0	0.00
Vegetables	4	1.04	50	1.14	6	1.84	0	0.00
Bean products	2	0.52	17	0.39	0	0.00	0	0.00
Egg and egg products	1	0.26	24	0.55	0	0.00	0	0.00
Fruits	1	0.26	2	0.05	0	0.00	0	0.00
Unknown	179	46.74	2092	47.74	137	42.02	1	100.00
Total	383	100.00	4382	100.00	326	100.00	1	100.00

a The cooked aquatic products included 106 seafood and 4 freshwater products.

### 3.6. Contributing factors

66.84% (256/383) of *Vibrio parahaemolyticus* outbreaks had at least one contributing factor. The most common contributing factors to outbreaks were improper food processing (67.57%, 173/256), cross contamination between raw and cooked food (39.06%, 100/256), and improper food storage (32.42%, 83/256). There were also factors such as contamination or deterioration of raw materials (10.55%, 27/256), contamination of equipment (10.55%, 27/256), contamination of personnel (4.30%, 11/256), and multiple factors were involved in 51.56% (132/256) of outbreaks. Outbreaks caused by Chinese cold dishes and meat and meat products often involved raw and cooked cross-contamination (52.08%, 25/48), while outbreaks caused by aquatic products often involved improper food processing (58.19%, 71/122).

The main contributing factors to outbreaks of cooked shrimp were improper food processing (54.84%, 17/31), cross contamination between raw and cooked food (22.58%, 7/31), improper storage (16.13, 5/31), contamination of raw materials (12.90%, 4/31), and contamination of equipment (9.68%, 3/31). For cooked shellfish, the main contributing factors were improper food processing (66.67%, 20/30), improper storage (20%, 6/30), contamination of raw materials (10%, 3/30), cross contamination between raw and cooked food (6.67%, 2/30). For cooked fish, the main contributing factors were improper food processing (65.22%, 15/23), cross-contamination between raw and cooked fish (39.13%, 9/23), improper storage (34.78%, 8/23), and equipment contamination (8.7%, 2/23).

### 3.7. Serotypes

Serotyping of 201 *Vibrio parahaemolyticus* outbreaks was performed, with serotype O3:K6 accounting for the largest proportion (63.18%, 127/201), followed by serotype O10:K4 (16.91%, 34/201) and O4:K8 (7.46%, 15/201) (Table 4). Serotype O3:K6 (81.94%, 118/144) was the dominant serotype in *Vibrio parahaemolyticus* outbreaks from 2010 to 2020, while serotype O10:K4 (57.89%, 33/57) was the dominant serotype from 2021 to 2022.

**Table 4:** Serotyping information of *Vibrio parahaemolyticus* outbreaks, 2010–2022, Zhejiang Province.

Serotype	Year			
	2010–2014	2015–2020	2021–2022	Total
O3:K6	19	99	9	127
O4:K8	1	11	3	15
O10:K4	0	1	33	34
Other	3	10	12	25
Total	23	121	57	201

## 4. Discussion

Our research has demonstrated that outbreaks caused by *Vibrio parahaemolyticus* had obvious seasonal characteristics, with most outbreaks occurring between July and September when the temperature was high, which was consistent with the conclusions of many studies (Iwamoto et al., 2010; Wang et al., 2015; Park et al., 2018). This is due to the mesothermal nature of *Vibrio parahaemolyticus*, resulting in higher concentrations of this bacterium in seafood or seawater during summer compared to winter (Cook et al., 2002; Han et al., 2012; Li et al., 2020). Previous studies has indicated that *Vibrio parahaemolyticus* outbreaks are primarily associated with the consumption of raw or undercooked seafood (Yeung and Boor, 2004). Our research also revealed that aquatic products (mainly seafood) were the foods responsible for most *Vibrio parahaemolyticus* outbreaks. Gooch et al. (2002) found that *Vibrio parahaemolyticus* in live oysters increased 50-fold and 790-fold after storage at 26°C for 10 and 24 h, respectively. Thus, even if the amount of *Vibrio parahaemolyticus* in freshly caught seafood is below the maximum level recommended by the FDA, its ability to multiply rapidly at ambient temperatures is likely to result in enough bacteria in the food to cause foodborne diseases (Yeung and Boor, 2004). In addition, according to a study in Zhejiang Province, the detection rate of *Vibrio parahaemolyticus* in raw/semi-raw seafood was as high as 32.52% (Mei et al., 2012). Therefore, the consumption of raw or undercooked seafood poses an extremely high risk. To reduce the risk of infection, on the one hand, we can implement measures to control the quantity of *Vibrio parahaemolyticus* in raw seafood, such as regulating the fishing temperature (McLaughlin et al., 2005), and promptly taking low-temperature preservation measures on freshly caught seafood to prevent its rapid multiplication to the level of infection. On the other hand, consumers should be reminded to avoid eating raw or undercooked seafood. It is noteworthy that in our study, raw aquatic products accounted for only 3.13% of outbreaks, while cooked aquatic products (28.72%) were the predominant food associated with outbreaks. Further analysis has revealed that the primary causes of outbreaks associated with cooked aquatic products are improper food processing and cross contamination between raw and cooked food. Therefore, targeted measures should be implemented to mitigate such outbreaks.

In our research, in addition to *Vibrio parahaemolyticus* outbreaks caused by seafood, freshwater products also caused some outbreaks (3.64%, 4/110) (Table 3). A study in Jiangsu, China (Chao et al., 2009) revealed that *Vibrio parahaemolyticus* was not only detected in seafood, but also in freshwater aquatic products and the strain of *Vibrio parahaemolyticus* found in freshwater aquatic products originated from seafood. Such cross-contamination primarily occurred at the point of sale, with markets, hotels and restaurants being the primary locations of concern. *Vibrio parahaemolyticus* has also been isolated from freshwater aquatic products in two recent studies (Wang et al., 2017; Li et al., 2019). In view of this, relevant authorities should strengthen the monitoring of *Vibrio parahaemolyticus* in freshwater products to reduce the infection caused by this pathogen.

In addition to *Vibrio parahaemolyticus* outbreaks associated with aquatic products, our research has also identified an increase in outbreaks linked to ready-to-eat foods in recent years. These foods are primarily Chinese cold dishes and cooked meat products that are not reheated prior to consumption, excluding any raw seafood. Studies also showed that *Vibrio parahaemolyticus* was isolated from some ready-to-eat foods (cooked meat, cold dishes, etc.) in China (Xie et al., 2016, 2020). Cross-contamination is the primary cause of *Vibrio parahaemolyticus* outbreaks associated with ready-to-eat foods. A study in Korea (Jung, 2018) reported a large outbreak of *Vibrio parahaemolyticus* due to cross contamination of squid for pancake and egg slices for kimbap using the same cutting board and knife, resulting in cross contamination of squid and kimbap rather than actual seafood consumption. We have also found similar causes of outbreaks. For example, some cooks used knives or cutting boards designated for raw food to prepare cooked meat products, and some used containers for raw food to hold cooked meat products. These non-standard procedures may result in cross-contamination between raw and cooked meat products, thereby leading to outbreaks. A survey conducted in China (Lu et al., 2015) on food handlers’ knowledge, attitudes and practices regarding food safety revealed that the majority of respondents lacked awareness about the maximum storage time for seafood at room temperature and common foodborne pathogens. Furthermore, when questioned about their practices, approximately one-fifth of respondents admitted to mixing raw and cooked food containers to varying degrees. To prevent contamination of ready-to-eat foods by *Vibrio parahaemolyticus*, food handlers should be knowledgeable about food safety and strictly adhere to safe food service practices. Given that *Vibrio parahaemolyticus* outbreaks occur more frequently in restaurants (57.96%), staff canteens (15.40%) and rural banquets (11.23%), it is imperative to implement enhanced supervision and specialized training programs for managers and new employees on seafood preparation, in order to effectively mitigate the risk of foodborne pathogens.

Our study found that aquatic products and ready-to-eat foods were the main food vehicles for *Vibrio parahaemolyticus* outbreaks in Zhejiang province. Furthermore, the outbreaks associated with aquatic products were primarily attributed to improper food processing (58.19%), whereas those linked to ready-to-eat foods were predominantly caused by cross-contamination between raw and cooked foods (52.08%). Therefore, in order to mitigate *Vibrio parahaemolyticus* outbreaks, priority should be given to addressing these two primary contributing factors. The catering units must strictly comply with the “Food Safety Operation Standard for Catering Services” (State Administration for Market Regulation, 2018) to ensure the safety of catering food. The regulations, which were issued by State Administration for Market Regulation and became effective on October 1st, 2018, pertain to the standards and fundamental specifications of catering establishments, food handling procedures, cleaning operations, utensil sanitation practices as well as takeaway delivery services. Additionally, it is recommended that the catering units adopt self-management practices and implement advanced food safety management systems such as Hazard Analysis and Critical Control Point (HACCP), Good Manufacturing Practices (GMP), etc. For household food handlers, the Five Keys to Safe Food (World Health Organization, 2012) must be followed, which involves proper handling techniques to eliminate harmful pathogens and prevent cross-contamination.

Furthermore, through 13 years of continuous surveillance, we have observed that the predominant serotype responsible for *Vibrio parahaemolyticus* outbreaks in Zhejiang Province from 2010 to 2020 was O3:K6. However, as of 2021, the main serotype has shifted to O10:K4, which is in line with the findings of screening for *Vibrio parahaemolyticus* in 11,166 stool samples from diarrhea patients in Huzhou, Zhejiang Province (Zhang et al., 2022). After the newly emerged serotype O10:K4 was isolated from outbreaks and sporadic cases in China, Thailand (Kazuhisa et al., 2023) also reported the first case of infection with *Vibrio parahaemolyticus* O10:K4 infection isolated from an inpatient with acute diarrhea and confirmed the emergence of the O10:K4 strain in Southeast Asia. Chinese researchers have suggested that O10:K4 is a clone of the pandemic strain O3:K6 (Zhang et al., 2022). The emergence of new serum variants poses a challenge to the prevention and control of outbreaks and epidemics of *Vibrio parahaemolyticus* in the future.

## 5. Conclusion

*Vibrio parahaemolyticus* outbreaks had obvious seasonal characteristics, and the majority of outbreaks occur in restaurants, staff canteens, and rural banquets. Aquatic products are the primary food vehicles for *Vibrio parahaemolyticus* outbreaks, while ready-to-eat foods are also susceptible to cross-contamination due to their lack of heating before consumption. To control *Vibrio parahaemolyticus* outbreaks, on the one hand, measures can be taken to reduce the concentration of this pathogen in raw aquatic products. On the other hand, adherence to good practices in food preparation and storage is crucial for preventing outbreaks caused by improper cooking and cross-contamination. Additionally, we should pay more attention to the newly emerged serotype O10:K4 in China and conduct further research. In the future, we will continue to strengthen the surveillance of foodborne disease outbreaks to provide data to support the prevention and control of foodborne diseases.

## Data availability statement

The data analyzed in this study is subject to the following licenses/restrictions: the data that support the findings of this study are available from the Foodborne Disease Outbreaks Surveillance System (https://sppt.cfsa.net.cn/goto), and these data are not publicly available. Requests to access these datasets should be directed to llchen@cdc.zj.cn.

## Author contributions

LC: conceptualization, project administration, and writing—original draft. YH: data curation. HZ: investigation. JW and XQ: methodology. RZ: supervision and validation. JC: writing—review and editing. All authors have read and agreed to the published version of the manuscript.

## Funding

This research was sponsored by Medical and Health Science and Technology Project of Zhejiang Province (Nos. 2022KY712 and 2022KY717).

## Conflict of interest

The authors declare that the research was conducted in the absence of any commercial or financial relationships that could be construed as a potential conflict of interest.

## Publisher’s note

All claims expressed in this article are solely those of the authors and do not necessarily represent those of their affiliated organizations, or those of the publisher, the editors and the reviewers. Any product that may be evaluated in this article, or claim that may be made by its manufacturer, is not guaranteed or endorsed by the publisher.
